# Prevalence and Associated Factors of Hypertension among Women in Southern Ghana: Evidence from 2014 GDHS

**DOI:** 10.1155/2022/9700160

**Published:** 2022-06-20

**Authors:** Cyprian Issahaku Dorgbetor, Kwamena Sekyi Dickson, Edward Kwabena Ameyaw, Kenneth Setorwu Adde

**Affiliations:** ^1^Ghana Health Service, Municipal Health Directorate, Techiman, Bono East Region, Ghana; ^2^University of Cape Coast, Department of Population and Health, College of Humanities and Legal Studies, Cape Coast, Ghana; ^3^Institute of Policy Studies and School of Graduate Studies, Lingnan University, Tuen Mun, Hong Kong

## Abstract

**Background:**

Hypertension, coupled with prehypertension and other hazards such as high blood pressure, is responsible for 8·5 million deaths from stroke, ischaemic heart disease, other vascular diseases, and renal disease worldwide. Hypertension is the fifth commonest cause of outpatient morbidity in Ghana. Some evidence have illustrated geographical variation in hypertension and it seems to have a heavy toll on women in southern Ghana compared to the north. This study seeks to determine the prevalence and associatedfactors of hypertension among women in southern Ghana using the most recent demographic and health survey (DHS) data set.

**Materials and Methods:**

This study used data of 5,662 women from the current DHS data from Ghana that was conducted in 2014. Data were extracted from the women's file of the 2014 Ghana DHS. The outcome variable of this current study was hypertension and it was measured by blood pressure, according to guidelines of the Joint National Committee Seven (JNC7). Multivariable binary logistic regression analyses were performed to establish the factors associated with hypertension at the individual and community levels.

**Results:**

Prevalence of hypertension among women in southern Ghana was 16%. Women aged 40–44 years (aOR = 8.04, CI = 4.88–13.25) and 45–49 years (aOR = 13.20, CI = 7.96–21.89] had the highest odds of hypertension relative to women aged 15–19 years. Women with two births (aOR = 1.45, CI = 1.01–2.07) and those with three births (aOR = 1.47, CI = 1.01–2.15) had a higher likelihood of being hypertensive. Greater Accra women had higher odds (aOR = 1.35, CI = 1.02–1.79) of being hypertensive relative to the reference category, women from the Western region. Women of Guan ethnicity had a lesser likelihood (aOR = 0.54, CI = 0.29–0.98) of being hypertensive. Women who engaged in agriculture had the least likelihood (aOR = 0.72, CI = 0.52–0.99) of being classified hypertensive compared to unemployed women.

**Conclusion:**

This study has revealed the prevalence of hypertension among women in southern Ghana. The associated factors include age, parity, region, and occupation. As a result, existing interventions need to be appraised in the light of these factors. Of essence is the need for Ghana Health Service to implement wide-embracing health promotion initiatives that accommodate the nutritional, exercise, and lifestyle needs of women in southern Ghana. Having more children is associated with higher propensity of hypertension and consequently, women need to limit childbearing to reduce their chances of being hypertensive. It will also be advisable for women in the Greater Accra region to have frequent hypertension screening, as women in the region exhibited higher hypertension prospects.

## 1. Background

Hypertension, coupled with prehypertension and other hazards, such as high blood pressure, is responsible for 8·5 million deaths from stroke, ischaemic heart disease, other vascular diseases, and renal disease worldwide [1]. In high-income countries, hypertension prevalence has declined while health systems have achieved treatment rates of about 80% and control rates of 60% [1]. Hypertension prevalence has generally increased while obesity, which is a risk factor for hypertension, decreased between 1990 and 2019 [1].

Hypertension reduction is a primary goal of the World Health Organization (WHO) global monitoring framework [2]. Besides, the expansion of universal health coverage and primary care in places with low rates of diagnosis, especially sub-Saharan Africa and South Asia, provide an opportunity for improving hypertension care [1].

Hypertension has been a key public health challenge in Ghana. It is known to be the fifth commonest cause of outpatient (OPD) morbidity in Ghana whilst at the same time, 63% of women with hypertension symptoms are oblivious of thier hypertension status [3]. Geographical variations in hypertension have been reported from some countries, including China and Gambia [4, 5].

In the case of Ghana, there is a seemingly wide difference in the Southern-Northern prevalence of hypertension with about half of the adults living in some parts of southern Ghana (including Eastern, Volta, Western, and Brong Ahafo regions) having the condition while about one quarter are either overweight or obese [6, 7]. The relatively lower prevalence in the north has been associated with the traditional way of living characterized by manual farming, housekeeping, and greater physical activity which people mostly take a walk or travel by bicycle [6–8]. The Southern-Northern differences in the prevalence of hypertension warrant empirical investigation since higher prevalence has persisted over the last four decades [6, 7].

It is based on this background that the current study aims to examine the prevalence and factors associated with hypertension among women in southern Ghana using the most recent Demographic and Health Survey (DHS) data set. Results from the study will contribute significantly towards the development of local and national level interventions and policies that will contribute to the prevention of hypertension across Ghana.

## 2. Methodology

### 2.1. Study Setting

**Figure 1:**
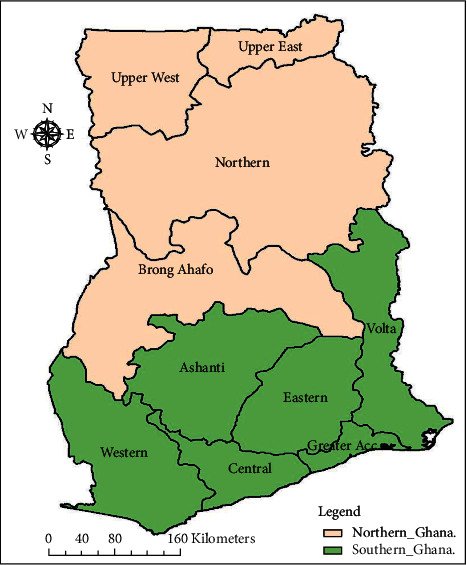
Southern Ghana.

The study was implemented in southern Ghana. Our study was based on 2014 Ghana Demographic and Health Survey (GDHS); and it is worth noting that prior to 2018 Ghana had ten administrative regions [9]. As stated by the Ghana Statistical Service, six out of the ten regions constitute southern Ghana and these are Western, Central, Greater Accra, Ashanti, Eastern, and Volta region as illustrated in green in Figure 1. One key distinction between southern and northern Ghana manifest in skeweness in development towards the south [10].

### 2.2. Sources of Data

This study used current DHS data of Ghana which was conducted in 2014 [11]. Specifically, data was pulled from the women's files of the DHS data set. DHS are national surveys in respective countries carried out approximately every five years in over 90 low- and middle-income countries in the world [12]. DHS concentrates on maternal and child health issues, including physical activity, non-communicable diseases, sexually transmitted infections, fertility, tobacco use, health insurance, and alcohol consumption. The survey categorically provides data to monitor the demographic and health profiles of included countries [12]. The sample for the present study consisted of women whose blood pressure were taken (aged 15–49 years) and had complete cases on all variables of interest (*N* = 5, 662). The DHS program permitted us access to the dataset after the evaluation of our concept note. The datasetis freely available and accessible to the public at https://www.measuredhs.com/.

### 2.3. Outcome Variable

The outcome variable of this study was hypertension, measured by blood pressure. For 2014 DHS, blood pressure was monitored and measured thrice and these followed the UA-767F/FAC (*A* & *D* Medical) blood pressure computation with a minimum of 10 minutes interval [10]. Hypertension status was determined by computing the average of the second and third measurements. It was in line with the calibration by similar empirical studies on hypertension that relied on DHS datasets [13]. Guided by the guidelines of the Joint National Committee Seven (JNC7), hypertension was computed as the average systolic blood pressure of ≥140 mmHg and/or an average diastolic blood pressure of ≥90 mmHg. Consequently, a hypertensive woman was identified as 1, and non-hypertensive women were identified otherwise “0.”

### 2.4. Explanatory Variables

Eleven explanatory variables were examined in the study. The selection of these variables was based on conceptual relevance and their significant association with the outcome variable based on findings from previous studies [14–17]. A number of studies have followed this approach [18, 19]. All variables were grouped into personal and community level variables based on the orderly nature of the dataset. The variables were determined based on their availability in the dataset, practical significance, and theoretical relevance for hypertension.

#### 2.4.1. Individual Variables

The individual level explanatory variables were wealth status, age, marital status, education, parity, occupation, and the consumption of salted fish in the last 24 hours. Age was recorded as 15–19, 20–24, 25–29, 30–34, 35–39, 40–44, and 45–49 years. Wealth status was categorized into poorest, poorer, middle, richer, and richest. Education was classified into four categories: no education, primary education, secondary education, and higher education. Occupation was identified unemployed, professional/clerical, sales/services, agricultural worker, and manual worker. Consumption of salted fish in the last 24 hours was coded as Yes and No.

#### 2.4.2. Community Variables

Four variables were selected at the community level. These are region, place of residence, ethnicity, and religion. The region was coded as Eastern, Western, Greater Accra, Central Volta, and Ashanti, since the study was limited to the Southern part of Ghana. Ethnicity was coded Ga/Dangme, Akan, Ewe, Guan, Mole-Dagbani, Gurma, Grusi, Mande, and others. Religion was identified as Islam, Christianity, and others. Place of residence was identified as urban and rural.

### 2.5. Data Analysis

Data were extracted, cleaned, and analyzed using Stata software version 13.0. Percentage was used to summarize the prevalence of hypertension among respondents. Cross-tabulation was adopted to examine the distribution of hypertension across explanatory variables. Results of cross-tabulation were displayed using percentages with their corresponding confidence intervals. Subsequently, multivariable binary logistic regression analysis was used to determine the factors related with hypertension. Three (3) models were built to examine the factors associated with hypertension. The first model (Model I) consisted of only individual-level variables. Model II was built to contain community-level variables, whilst in Model III, all explanatory variables were combined to examine their association with hypertension. Results of the study were presented using adjusted odds ratio (aOR) with their respective 95% confidence intervals. The women's sample weights (v005/1,000,000) were utilized to achieve unbiased estimates, and the Stata survey command “svy” was used to correct for the data's complex sampling structure in the chi-square test and regression analyses, as recommended by DHS.

### 2.6. Model Fit and Specifications

We evaluated the fitness of all models with Akaike's information criterion (AIC) and Bayesian information criterion (BIC). The presence of multicollinearity between independent variables was checked before fitting these models. The variance inflation factor (VIF) test revealed the absence of high multicollinearity between variables (Mean VIF = 3.01).

### 2.7. Ethical Approval

This study included participation of human subjects; however, the authors of this manuscript were not directly involved in data collection processes. According to the final report of 2014 GDHS, the survey protocol, including biomarker collection, was reviewed and approved by the Ghana Health Service' Ethical Review Committee and the Institutional Review Board of ICF International [10]. For every research participant, either written or verbal consent was obtained.

## 3. Results

### 3.1. Background Characteristics

The study revealed that 17.3% of women were between 25–29 years. The majority of the women had secondary education (63%), were Christians (87.1%), were from the Akan ethnic group (58.1%), and were from urban residences (59.5%) (see Table 1). A higher proportion of women were married (38.9%), had zero birth (32.2%), within the richest wealth status (28.6%), were in sales/service occupation (43.8%), and from the Greater Accra region (26.2%) (see Table 1).

### 3.2. Hypertension Prevalence by Socio-Demographic Characteristics

The prevalence of hypertension was 16%. Four in ten women aged 45–49 were hypertensive. A higher proportion of women of richest wealth index (20%) were hypertensive. Twenty-one percent of women with no education (21%) were seen to be hypertensive (see Table 2). A higher proportion of women from urban residences (18%) were also hypertensive (see Table 2).

### 3.3. Multivariate Logistic Regression

From the three models, Model 1 was the best fit model (BIC = 4544.841, AIC = 4392.086). However, we reported findings from the second-best fit model (Model 3) because it is the only complete model (BIC = 4687.386, AIC = 4401.800). Age, parity, region, ethnicity, and occupation had significant association with hypertension among women in southern Ghana. The variance inflation factor (VIF) test revealed that there is no multicollinearity among the socio-demographic variables (Min = 1.05, Max = 13.14, and Mean VIF = 3.01). Older women had a higher likelihood of hypertension compared to younger women. For instance, women aged 40–44 years (aOR = 8.04, CI = 4.88–13.25) and those 45–49 years (aOR = 13.20, CI = 7.96–21.89) revealed higehr odds compared to women aged 15–19 (see Table 3).

Women with two births (aOR = 1.45, CI = 1.01–2.07) and those with three births (aOR = 1.47, CI = 1.01–2.15) had higher likelihood of being hypertensive compared to those with zero birth. Greater Accra women had higher odd (aOR = 1.35, CI = 1.02–1.79) of being hypertensive compared to the reference category, thus women from the Western region (see Table 3). Women of Guan ethnicity had lesser likelihood (aOR = 0.54, CI = 0.29–0.98) of being hypertensive compared to women with the Akan ethnic group. Women who engagedin agricultural work had lesser likelihood (OR = 0.72, CI = 0.52–0.99) of being hypertensive compared to unemployed women (see Table 3).

### 3.4. Discussion

The objective of the study was to investigate the magnitude of hypertension in women across southern Ghana and underlying socio-demographic factors, as previous studies have not prioritized this inquiry. Age, parity, region, ethnicity, and occupation had significant association with hypertension among women in southern Ghana. The total prevalence was 16% which is relatively lower than the reported prevalence for women in the middle belt of Ghana, which was 28.1% [20]. Relatedly, a systematic review has revealed that urban residents in Ghana tend to have a higher prevalence. Considering the high urbanization rate of southern Ghana relative to other parts of the country, coupled with the variations in physical and dietary patterns as well as migration induced psychosocial distress, the hypertension situation in southern Ghana may rise if effective public health interventions are not implemented [21–23]. The finding is suggestive that southern Ghana may require well-tailored and effective anti-hypertension interventions to overcome the condition. Besides, health promotion interventions can be rolled out through various media platforms across southern Ghana.

Women who were older had a higher probability of hypertension relative to younger women. High inclination of older women to hypertension is extensively reported in the literature from Ghana [24, 25], as well as other low and middle-income countries (LMICs) [26, 27] and advanced countries like USA [28]. It is worth noting that in the global west (specifically USA and Europe), between one quarter and one-third of adult populations are hypertensive [29, 30]. Our finding is consistent with the existing literature and aging-induced arterial stiffening has been identified to increase the likelihood of hypertension among persons who are advanced in age [31, 32].

Women with two or three births had the highest probability of being hypertensive relative to those without birth. Contrary to this, in Ethiopia, a case-control study revealed that women at parity zero had about seven times higher likelihood of hypertension compared with those with one or higher parity or birth [33]. Several factors may account for the discrepancy in findings. For instance, whilst the present study employed a cross-sectional design, the Ethiopian based study was a facility-based retrospective unmatched case-control which was organized in a single south-western based referral hospital. Further, considering the strong correlation between diet, lifestyle, and hypertension, it is unlikely that different groups in distinct environmental contexts will have equal propensities of hypertension in their lifetime [34, 35]. Our finding here accentuates the need for maternity healthcare providers to consider hypertension education as a critical component of reproductive health services, with emphasis on antenatal care (ANC) and postnatal care (PNC) in southern parts of Ghana.

Greater Accra women experienced increased odds of being hypertensive relative to the women in the Western region. Guan women had a lesser probability of being hypertensive relative to women of Akan ethnicity. These plausibly imply that some ethnic- and region-based peculiar characteristics dictate women's chances of hypertension. For instance, production and distribution of fruits are uneven across the various regions of Ghana and these could disadvantage residents of some regions whilst making it easy for others to get access to fruits, hence having less likelihood of hypertension [36, 37].

Lastly, women who engaged in agricultural work had lesser probability of hypertension relative to unemployed women. Considering extensive documentation on the inverse relationship between vigorous/intensive work and hypertension, this finding is anticipated [37, 38]. Subsequently, unemployed women require behavioral change and continuous engagement in at least moderate exercises in order to subside their chances of hypertension.

### 3.5. Strengths and Limitations

It quite interesting to note that this is the first study to investigate the prevalence of hypertension and underlying conditions among women in southern Ghana. Besides, appropriate statistical techniques have been applied. One limitation of the study is that it was limited to females. We focused on femelaes because consistent evidence have revealed that women bear the greatest risk of hypertension relative to men [39, 40]. Second, a cross-sectional study design was employed by the study. Due to this, causal inference cannot be drawn.

## 4. Conclusion

This study aimed at expanding the frontiers of knowledge on hypertension by investigating the prevalence and underlying factors among women in southern Ghana. From the findings, it will be useful for the Ghana Health Service to implement wide-embracing health promotion initiatives that accommodate the nutritional, exercise, and lifestyle needs of women in southern Ghana. The study has revealed a number of associates of hypertension including age, parity, region, ethnicity, and occupation; hence, existing interventions need to be appraised in the light of these identified factors. Finally, fruit consumption habits and maintenance of the normal body weight ought to be encouraged among women in southern Ghana.

## Figures and Tables

**Table 1:** Background characteristics of respondents.

Variable	Frequency (*n* = 5.662)	Percentage (%)
Hypertension		
Non hypertensive	4744	83.8
Hypertensive	918	16.2
Age (years)		
15–19 years	922	16.3
20–24 years	972	17.2
25–29 years	981	17.3
30–34 years	837	14.8
35–39 years	791	14.0
40–44 years	636	11.2
45–49 years	523	9.2
Wealth index		
Poorest	325	5.7
Poorer	949	16.8
Middle	1273	22.5
Richer	1496	26.4
Richest	1620	28.6
Level of education		
No education	678	12.0
Primary	991	17.5
Secondary	3575	63.1
Higher	418	7.4
Ethnicity		
Akan	3287	58.0
Ga/Dangme	562	9.9
Ewe	973	17.2
Guan	108	1.9
Mole-dagbani	361	6.4
Grusi	101	1.8
Gurma	116	2.1
Mande	28	0.5
Others	126	2.2
Religion		
Islam	534	9.4
Christian	4930	87.1
Others	198	3.5
Parity		
Zero birth	1823	32.2
One birth	819	14.5
Two births	823	14.5
Three births	694	12.3
Four births or more	1503	26.6
Marital status		
Single	1921	33.9
Married	2203	38.9
Living with partner	873	15.4
Widowed	146	2.6
Separated	519	9.2
Occupation		
Unemployed	1310	23.1
Professional/clerical	450	7.9
Sales/services	2479	43.8
Agricultural worker	704	12.4
Manual worker	719	12.7
Salted fish in the last 24 hrs		
No	3539	62.5
Yes	2123	37.5
Place of residence		
Urban	3370	59.5
Rural	2292	40.5
Region		
Western	814	14.4
Central	732	12.9
Greater accra	1482	26.2
Volta	563	9.9
Eastern	680	12.0
Ashanti	1393	24.6

**Table 2:** Hypertension prevalence by socio-demographic characteristics.

Socio-demographic characteristics	Hypertensive		*X* ^2^; *pvalue*
	Yes *n* (%)	No *n* (%)	
Age			*X* ^2^ = 545.90; *p* < 0.001
15–19	39 (4%)	938 (96%)	
20–24	55 (6%)	910 (94%)	
25–29	97 (10%)	850 (90%)	
30–34	125 (15%)	711 (85%)	
35–39	179 (23%)	591 (77%)	
40–44	187 (29%)	455 (71%)	
45–49	209 (40%)	316 (60%)	
Wealth index			*X* ^2^ = 27.832; *p* ≤ 0.001
Poorest	53 (13%)	350 (87%)	
Poorer	144 (13%)	964 (87%)	
Middle	206 (15%)	1164 (85%)	
Richer	208 (15%)	1168 (85)	
Richest	280 (20%)	1125 (80%)	
Level of education			*X* ^2^ = 21.13; *p* ≤ 0.001
No education	151 (21%)	585 (79%)	
Primary	167 (16%)	865 (84%)	
Secondary	502 (14%)	3014 (86%)	
Higher	71 (19%)	307 (81%)	
Ethnicity			*X* ^2^ = 8.36; *p* ≤ 0.399
Akan	515 (16%)	2728 (84%)	
Ga/dangme	86 (17%)	416 (83%)	
Ewe	181 (17%)	901 (83%)	
Guan	14 (11%)	115 (89%)	
Mole-dagbani	46 (14%)	283 (86%)	
Grusi	12 (13%)	77 (87%)	
Gurma	15 (11%)	125 (89%)	
Mande	5 (20%)	20 (80%)	
Others	17 (14%)	106 (86%)	
Religion			*X* ^2^ = 1.04; *p* ≤ 0.593
Islam	76 (16%)	392 (84%)	
Christian	775 (16%)	4197 (84%)	
Others	40 (18%)	182 (82%)	
Parity			*X* ^2^ = 230.62; *p* ≤ 0.001
Zero birth	119 (7%)	1683 (93%)	
One birth	98 (12%)	725 (88%)	
Two births	154 (19%)	663 (81%)	
Three births	142 (21%)	525 (79%)	
Four births or more	378 (24%)	1175 (76%)	
Marital status			*X* ^2^ = 233.28; *p* ≤ 0.001
Single	128 (7%)	1809 (93%)	
Married	465 (21%)	1720 (79%)	
Living with partner	124 (14%)	755 (86%)	
Widowed	46 (31%)	102 (69%)	
Separated	128 (25%)	385 (75%)	
Occupation			*X* ^2^ = 76.65; *p* ≤ 0.001
Unemployed	123 (9%)	1270 (91%)	
Professional/clerical	81 (19%)	351 (81%)	
Sales/services	455 (19%)	1898 (81%)	
Agricultural worker	130 (16%)	668 (84%)	
Manual worker	102 (15%)	584 (85%)	
Salted fish in the last 24 hrs			*X* ^2^ = 0.08; *p* ≤ 0.774
No	549 (16%)	2964 (84%)	
Yes	342 (16%)	1807 (84%)	
Place of residence			*X* ^2^ = 17.08; *p* ≤ 0.001
Urban	558 (18%)	2631 (82%)	
Rural	333 (13%)	2140 (87%)	
Region			*X* ^2^ = 29.95; *p* ≤ 0.001
Western	141 (14%)	884 (86%)	
Central	126 (13%)	809 (87)	
Greater Accra	204 (21%)	789 (79%)	
Volta	125 (16%)	666 (84%)	
Eastern	118 (13%)	774 (87%)	
Ashanti	177 (17%)	849 (83%)	
Total	891 (16%)	4771 (84%)	

**Table 3:** Multivariate logistic regression.

Variables	Model 1	Model 2	Model 3
Individual variables			
Age (years)			
15–19 years	Ref		Ref
20–24 years	1.28 [0.83–1.99]		1.31 [0.84–2.06]
25–29 years	2.09 [1.34–3.25]^*∗∗∗*^		2.22 [1.40–3.51]^*∗∗∗*^
30–34 years	3.12 [1.97–4.94]^*∗∗∗*^		3.29 [2.03–5.34]^*∗∗∗*^
35–39 years	5.41 [3.40–8.61]^*∗∗∗*^		5.67 [3.47–9.26]^*∗∗∗*^
40–44 years	7.56 [4.71–12.12]^*∗∗∗*^		8.04 [4.88–13.25]^*∗∗∗*^
45–49 years	12.33 [7.64–19.89]^*∗∗∗*^		13.20 [7.96–21.89]^*∗∗∗*^
Level of education			
No education	Ref		Ref
Primary	1.01 [0.78–1.31]		0.97 [0.75–1.27]
Secondary	0.96 [0.77–1.22]		0.94 [0.73–1.19]
Higher	1.17 [0.81–1.71]		1.06 [0.68–1.64]
Wealth index			
Poorest	Ref		Ref
Poorer	0.98 [0.69–1.39]		0.96 [0.67–1.37]
Middle	1.23 [0.87–1.74]		1.09 [0.75–1.58]
Richer	1.34 [0.94–1.90]		1.05 [0.69–1.59]
Richest	1.92 [1.34–2.75]^*∗∗∗*^		1.39 [0.89–2.17]
Parity			
Zero birth	Ref		Ref
One birth	1.14 [0.82–1.62]		1.16 [0.82–1.63]
Two births	1.44 [1.01–2.06]^*∗*^		1.45 [1.01–2.07]^*∗∗*^
Three births	1.47 [1.01–1.14]^*∗*^		1.47 [1.01–2.15]^*∗∗*^
Four births or more	1.29 [0.89–1.86]		1.31 [0.91–1.89]
Marital status			
Single	Ref		Ref
Married	0.97 [0.7–1.35]		0.99 [0.72–1.38]
Living with partner	0.93 [0.65–1.32]		0.95 [0.67–1.35]
Widowed	1.10 [0.68–1.79]		1.11 [0.68–1.81]
Separated	1.16 [0.81–1.69]		1.17 [0.81–1.70]
Salted fish in the last 24 hrs			
No	Ref		Ref
Yes	1.02 [0.87–1.19]		1.06 [0.90–1.24]
Community variables			
Region			
Western		Ref	Ref
Central		0.93 [0.72–1.21]	0.87 [0.66–1.15]
Greater accra		1.43 [1.09–1.86]^*∗∗*^	1.35 [1.02–1.79]^*∗*^
Volta		1.28 [0.91–1.81]	1.12 [0.78–1.62]
Eastern		0.96 [0.73–1.25]	0.89 [0.67–1.19]
Ashanti		1.19 [0.93–1.53]	1.13 [0.87–1.47]
Ethnicity			
Akan		Ref	Ref
Ga/Dangme		0.94 [0.71–1.25]	0.87 [0.65–1.17]
Ewe		0.98 [0.76–1.27]	1.04 [0.79–1.36]
Guan		0.61 [0.34–1.09]	0.54 [0.29–0.98]^*∗*^
Mole-dagbani		0.69 [0.48–1.02]	0.89 [0.59–1.34]
Grusi		0.69 [0.37–1.32]	0.78 [0.40–1.52]
Gurma		0.53 [0.29–0.94]^*∗*^	0.67 [0.36–1.23]
Mande		1.09 [0.39–3.08]	1.39 [0.46–4.22]
Others		0.63 [0.35–1.12]	0.75 [0.41–1.37]
Religion			
Islam		Ref	Ref
Christian		0.78 [0.56–1.09]	0.83 [0.58–1.18]
Others		0.94 [0.59–1.52]	0.82 [0.49–1.36]
Place of residence			
Urban		Ref	Ref
Rural		0.77^*∗∗*^ [0.65–0.92]	0.93 [0.75–1.15]
Occupation			
Unemployed		Ref	Ref
Professional/clerical		2.17 [1.59–2.95]^*∗∗∗*^	1.06 [0.72–1.57]
Sales/services		2.36 [1.91–2.93]^*∗∗∗*^	0.94 [0.73–1.21]
Agricultural worker		2.36 [1.79–3.13]^*∗∗∗*^	0.72 [0.52–0.99]^*∗*^
Manual worker		1.72 [1.29–2.29]^*∗∗∗*^	0.76 [0.55–1.03]
Random effect result			
*p*-value	0.001	0.001	0.001
BIC	4544.841	4983.376	4687.386
AIC	4392.086	4843.904	4401.800

Computed from 2014 Ghana demographic and health survey; *Ref* reference category; *BIC* Bayesian information criterion; *AIC* Akaike information criterion ^*∗*^*p* < 0.05; ^*∗∗*^*p* < 0.01; ^*∗∗∗*^*p* < 0.001.

## Data Availability

Data used for the study are freely available to the public at https://dhsprogram.com/data/available-datasets.cfm.

## Abbreviations

aOR: Adjusted odds ratio
CI: Confidence interval
CrI: Credible interval
DIC: Deviance information criterion
EA: Enumeration areas
GDHS: Ghana demographic and health survey
GSS: Ghana statistical service
IRB: Institutional review board
MCMC: Markov chain monte carlo
MOR: Median odds ratio
NHIA: National health insurance authority
NHIS: National health insurance scheme
OR: Odds ratio
PHC: Population and housing census
SSA: Sub-Saharan Africa
VIF: Variance inflation factor.
